# Effects of Dietary Nitrate and Caffeine on End Power and Work Above End Power During a 3 min All-Out Test in Trained Male Cyclists

**DOI:** 10.3390/nu18091463

**Published:** 2026-05-02

**Authors:** Anthony M. Hagele, Kyle L. Sunderland, Petey W. Mumford, Chad M. Kerksick

**Affiliations:** 1 Exercise and Performance Nutrition Laboratory, College of Science, Technology, and Health, Lindenwood University, St. Charles, MO 63301, USA; 2 Department of Biomedical Sciences, College of Osteopathic Medicine, Pacific Northwest University of Health Sciences, Yakima, WA 98901, USA

**Keywords:** dietary nitrate, caffeine, critical power, 3 min all-out test, beetroot juice, cycling performance, ergogenic aids

## Abstract

Background: The purpose of this study was to examine the effects of acute dietary nitrate (NO_3_^−^) and caffeine (CAF) supplementation on end power (EP) and work performed above EP (WEP) in trained male cyclists during a 3 min all-out test (3MT) on a cycle ergometer. Methods: Fifteen healthy, trained male cyclists (28.5 ± 5.3 years, 79.2 ± 9.1 kg, VO_2_peak 55.2 ± 5.6 mL·kg^−1^·min^−1^) completed four exercise trials in a randomized, double-blind, placebo-controlled, crossover study design separated by 3–7 days. The four experimental conditions were placebo beverage (nitrate-depleted) + placebo capsule, nitrate-rich beetroot juice + placebo capsule (BR), placebo beverage + caffeine capsule (CAF), and nitrate-rich beetroot juice + caffeine capsule (BR + CAF). Participants consumed nitrate-rich beetroot juice (~13 mmol NO_3_^−^) or nitrate-depleted placebo three hours before exercise, and caffeine (5 mg∙kg^−1^) or maltodextrin placebo one hour before testing. EP and WEP were determined from the 3MT. Secondary outcomes included peak and mean power output. Data were analyzed using a repeated-measures ANOVA with repeated measures on condition. A *p*-value of 0.05 was used to determine statistical significance. Effect size was evaluated using partial eta squared. Results: No significant effect of condition was observed for EP (*p* = 0.401, ηp^2^ = 0.056), WEP (*p* = 0.580, ηp^2^ = 0.048), peak power (*p* = 0.642, ηp^2^ = 0.046), mean power (*p* = 0.212, ηp^2^ = 0.108), or total work (*p* = 0.217, ηp^2^ = 0.107). Conclusions: No statistically significant differences between conditions were detected under the conditions of the present study.

## 1. Introduction

Dietary nitrate (NO_3_^−^) has become a commonly implemented ergogenic strategy among endurance athletes [1,2]. Naturally occurring sources of NO_3_ include green leafy vegetables and beetroot juice, with supplementation commonly provided via nitrate-rich beetroot juice or inorganic nitrate salts [3]. Following ingestion, NO_3_ is reduced to nitrite and subsequently nitric oxide (NO) through the enterosalivary nitrate–nitrite–NO pathway, where it acts peripherally as a signaling molecule involved in the regulation of blood flow, muscle contractility, glucose homeostasis, and mitochondrial respiration [4]. These physiological effects have been associated with reduced oxygen cost of submaximal exercise and improved exercise tolerance, translating to improvements in performance, particularly in tasks of moderate-to-severe intensity and exercise durations in the ~5–30 min range in young, healthy, physically active individuals [1,5,6]. However, meta-analyses indicate that performance benefits may be attenuated in aerobically trained individuals, and substantial inter-individual variability in responsiveness has been reported [7,8]. Despite this evidence, further research is required to clarify optimal dosing strategies, exercise contexts, and the potential for NO_3_ to interact with other ergogenic aids [5].

Caffeine is one of the most extensively studied ergogenic aids, with well-documented effects on endurance, strength, and recovery [9,10]. Its primary mechanism of action is central in nature, acting as an adenosine receptor antagonist to increase central drive, motor unit recruitment, and pain tolerance [11,12,13]. These effects may be particularly relevant during severe-intensity exercise, where tolerance to discomfort and the ability to sustain high motor output are critical determinants of performance [14]. Importantly, these centrally mediated effects are mechanistically distinct from the predominantly peripheral actions attributed to nitrate-derived nitric oxide, and it cannot be assumed that co-ingestion will result in additive benefits. Although caffeine and nitrate independently demonstrate ergogenic efficacy, limited research has examined their use within the same experimental model on exercise performance [5,15].

A key determinant of exercise performance is the concept of critical power (CP) and the associated curvature constant, *W*′, which represents the finite amount of work that can be performed above CP. Together, these parameters describe the hyperbolic relationship between power output and time to exhaustion, with CP demarcating the boundary between the ‘heavy’ and ‘severe’ exercise intensity domains and representing the highest sustainable work rate at which a metabolic steady state can be maintained [16,17]. One established method of estimating the CP and *W*′ is through the 3 min all-out cycling test (3MT), which provides estimates that are commonly interpreted as proxies for CP and *W*′ in the form of end power (EP) and work completed above end power (WEP) [17]. Although CP and related performance indices have been shown to respond to dietary nitrate or caffeine supplementation when administered independently, no studies to date have examined their combined effects on EP or WEP when administered within the same experimental model [18,19].

Given that dietary nitrate and caffeine each have demonstrated ergogenic effects when consumed independently and are thought to influence performance through distinct central and peripheral physiological mechanisms, it is therefore important to examine their effects within a controlled experimental model, particularly in the context of short-duration, high-intensity exercise. Therefore, the purpose of this study was to examine the effects of dietary nitrate and caffeine supplementation on performance outcomes derived from a 3MT, specifically EP and WEP, as surrogate measures of critical power and *W*′ in trained male cyclists. It was hypothesized that caffeine and nitrate would improve EP and WEP relative to placebo.

## 2. Materials and Methods

### 2.1. Experimental Design

Fifteen trained male cyclists participated in this double-blind, placebo-controlled, crossover study. The study protocol was approved by the Institutional Review Board at Lindenwood University (IRB-20-170; approval date: 1 October 2020), and all procedures were conducted in accordance with the ethical standards of the Declaration of Helsinki. Each participant completed six laboratory visits, consisting of two familiarization sessions followed by four supplementation trials. During the first visit, participants were first informed of all study procedures and provided written informed consent prior to participation. Height and body mass were then measured, followed by an incremental cycling exercise test to determine VO_2_peak, maximal power output (W_max_), and gas exchange threshold (GET). Participants also completed a familiarization trial of the 3MT. At least 48 h later, participants returned to the laboratory to complete a second 3MT familiarization trial. Familiarization trials were performed to minimize potential learning effects and improve test reliability. During visits three through six, participants completed four identical experimental trials under different supplementation conditions. All visits were conducted in the morning between 0600 and 1000 h. Prior to each experimental visit, participants fasted for 8–10 h and abstained from caffeine for 24 h. Participants were also instructed to avoid nitrate-rich foods for 24 h prior to testing and were provided with a list of common high-nitrate foods to facilitate compliance. To minimize the influence of recent physical activity, participants refrained from strenuous exercises for 48 h and from all exercise for 24 h prior to each visit. In addition, participants were instructed to avoid the use of antibacterial mouthwash throughout their participation to preserve oral bacteria involved in nitrate reduction. Participants were further instructed to replicate their dietary intake during the 24 h preceding each experimental visit with respect to total energy intake and macronutrient composition. Adherence to these instructions was based on self-report. Upon arrival at the laboratory, participants ingested either nitrate-rich beetroot juice or nitrate-depleted beetroot juice. After 120 min, participants consumed a capsule containing either caffeine or a placebo. Participants then rested for approximately 60 min before completing the 3MT. Experimental trials were separated by 3–7 days. This trial was registered at ClinicalTrials.gov (NCT07501806). Registration was completed retrospectively, as the study was initially designed and approved as a university-based investigation without a requirement for prospective trial registration.

### 2.2. Participants

Participant characteristics are presented in Table 1. All participants were healthy and free from known metabolic or cardiovascular disease. Participants were classified as trained cyclists using predefined training-history and objective performance criteria, consistent with published subject-classification guidelines [20]. Eligibility required a minimum of one year of cycling experience, regular cycling training performed ≥3 days per week, and ≥5 h per week. In addition, participants were required to meet at least one of the following performance criteria: (1) relative VO_2_peak within or approaching the trained range (approximately 55–64.9 mL∙kg^−1^∙min^−1^), or (2) peak power output expressed in absolute (≈320–380 W) or relative terms (≈4.6–5.5 W∙kg^−1^) consistent with trained performance standards [20]. These criteria align with established classifications of trained but non-elite cyclists.

### 2.3. Supplementation Protocol

Participants completed four supplementation conditions in a random order.

(I) Placebo beverage with placebo capsule (PLA).
(II) Nitrate-rich beetroot juice with a placebo capsule (BR).
(III) Placebo beverage with caffeine capsule (CAF).
(IV) Nitrate-rich beetroot juice with caffeine capsule (BR + CAF).

The treatment order was randomized and counterbalanced across participants using a computerized random number generator. For each experimental trial, participants consumed two concentrated beetroot juice shots (Beet-It Sport, James White Drinks Ltd., Ashbocking, UK; 70 mL per shot) providing ~13 mmol of nitrate or a nitrate-depleted placebo beverage (<0.1 mmol nitrate) from the same manufacturer, administered 180 min prior to the 3MT. Sixty minutes prior to the 3MT, participants ingested a capsule containing caffeine (5 mg∙kg^−1^ body mass) or a volume-matched maltodextrin placebo. All capsules were identical in size, appearance, and color to maintain blinding. The timing and dosages of nitrate and caffeine were selected in accordance with current evidence-based recommendations, with dietary nitrate provided 2–3 h prior to exercise [1] and caffeine administered at 3–6 mg∙kg^−1^ body mass approximately 60 min prior to exercise [9].

### 2.4. Dietary Control

Prior to each testing visit, participants completed a 24 h food record using the Automated Self-Administered 24 h Dietary Assessment Tool (ASA24; National Cancer Institute, Bethesda, MD, USA) to document habitual dietary intake. Participants were instructed to replicate their caloric intake and macronutrient distribution for the 24 h preceding each visit to minimize between-trial dietary variability. No specific dietary prescriptions were provided, and participants were instructed to maintain their habitual dietary patterns across all conditions.

### 2.5. Determination of W_max_, VO_2_peak and Gas Exchange Threshold

Participants completed a peak oxygen consumption test (VO_2_peak) using indirect calorimetry on a ParvoMedics TrueOne metabolic cart (ParvoMedics, Sandy, UT, USA) interfaced with a motorized cycle ergometer (Lode Excalibur Sport, Groningen, The Netherlands). Participants completed a standardized 5 min warm-up at 100 W, followed by a 5 min seated rest period. Following 30 s of unloaded baseline pedaling, participants performed a continuous ramp protocol beginning at 100 watts (W) with power output increasing by 1 W every 2 s (equivalent to 30 W·min^−1^) until volitional exhaustion. Participants were instructed to maintain a self-selected cadence between 60 and 100 rpm, determined during the warm-up period. The test was terminated when cadence fell more than 10 rpm below the self-selected cadence for longer than 10 s despite strong verbal encouragement.

Pulmonary gas exchange was measured breath-by-breath throughout the test and averaged over 10 s intervals. VO_2_peak was defined as the highest ten-second average VO_2_ achieved during the test and was considered valid if at least two of the following criteria were met:

- Respiratory exchange ratio ≥ 1.05.
- Heart rate within 10 beats∙min^−1^ of age-predicted maximal heart rate (HR_max_ = 208 − [0.7 × age]).
- A plateau in VO_2_ is defined as a change of <250 mL∙min^−1^ over two consecutive 10 s sampling intervals.
- Maximal power output (W_max_) was defined as the highest power output achieved during the incremental test.

Gas exchange threshold (GET) was determined using a combination of standard criteria, including:

1. The first disproportionate increase in carbon dioxide production (VCO_2_) relative to VO_2_ (V-slope method).
2. An increase in ventilatory equivalent for oxygen (*V_E_*/VO_2_) without a corresponding increase in ventilatory equivalent for carbon dioxide (*V_E_*/VCO_2_).
3. An increase in end-tidal oxygen pressure without a decrease in end-tidal carbon dioxide pressure.

GET was identified by visual inspection of these parameters by two independent investigators. In cases where agreement could not be reached, a third investigator reviewed the data, and a consensus was reached.

### 2.6. 3 min All-Out Test

EP and WEP were derived using a 3MT, following established procedures described by Vanhatalo et al. [21]. Prior to each trial, participants completed a standardized 5 min warm-up at 100 W, followed by a 5 min seated rest period. Each trial began with 3 min period of unloaded cycling at the participant’s self-selected cadence. Participants were instructed to increase their cadence to approximately 110 rpm during the final five seconds of the baseline period, after which they performed a 3 min maximal effort. Resistance during the all-out phase was set in linear mode such that participants achieved a power output corresponding to 50% of the difference between power at GET and the power output at VO_2_peak (i.e., GET + (50% × Δ), where Δ represents the difference between GET and VO_2_peak) upon reaching their self-selected cadence. Participants were instructed to maintain the highest possible cadence throughout the test and received strong verbal encouragement. To minimize pacing behavior, participants were not informed of elapsed time. Pulmonary gas exchange was measured breath-by-breath throughout the test using a metabolic cart (ParvoMedics TrueOne, Sandy, UT, USA) to verify that a maximal physiological response was elicited, with VO_2_peak recorded as the highest 30 s average attained during the 3 min effort. EP was calculated as the mean power output during the final 30 s of the test, and WEP was calculated as the total work performed above EP across the 3MT. In addition, secondary performance variables included peak power output, mean power output, total work, and mean power output averaged in 30 s intervals (0–30 s, 0–60 s, 0–90 s, 0–120 s, and 0–150 s).

### 2.7. Statistical Analysis

All data are presented as means ± standard deviations. Statistical analyses were performed using IBM SPSS Statistics for Windows, version 29 (IBM Corp., Armonk, NY, USA). A formal a priori sample size calculation was not conducted prior to participant recruitment. To provide context for the study design, a retrospective sample size estimation was performed using G*Power (version 3.1; Heinrich Heine University, Düsseldorf, Germany) for a repeated-measures ANOVA (within-factors). Assuming a moderate effect size (f = 0.25), an alpha level of 0.05, a desired statistical power of 0.80, four repeated conditions, and a correlation among repeated measures of 0.5, the estimated required sample size was 24 participants (actual power = 0.81).

A one-way repeated-measures ANOVA was used to examine the effect of condition (PLA, BR, CAF, BR + CAF) on primary outcomes (EP and WEP) and secondary variables, including peak power, mean power, total work, time-specific power outputs, and physiological responses. To assess potential order effects, a repeated-measures ANOVA was conducted with period (trials 1–4) included as a within-subject factor for each outcome variable. When a significant main effect was observed, pairwise comparisons were conducted using Bonferroni-adjusted post hoc tests. Assumptions of normality and sphericity were assessed using Shapiro–Wilk and Mauchly’s tests, respectively. Where the assumption of sphericity was violated, Greenhouse–Geisser corrections were applied. Effect sizes were calculated using partial eta squared (ηp^2^). Values of 0.01, 0.06, and 0.14 were interpreted as small, medium, and large effects, respectively [22]. In addition to null hypothesis significance testing, 95% confidence intervals were calculated for primary outcomes to provide an estimate of precision. Statistical significance was accepted at *p* ≤ 0.05. Figures were generated using GraphPad Prism (Version 10.4.1; GraphPad Software, San Diego, CA, USA).

## 3. Results

### 3.1. Reliability of Primary Performance Outcomes

To reduce learning effects and maximize reliability between testing conditions, each study participant completed two familiarizations. Test–retest reliability between familiarization trials was assessed using a two-way mixed-effects intraclass correlation coefficient with absolute agreement (ICC [3, 1]). Reliability was good for EP (ICC = 0.805; 95% CI: 0.552–0.922), WEP (ICC = 0.863; 95% CI: 0.671–0.946), and peak power (ICC = 0.805; 95% CI: 0.551–0.922), and excellent for mean power (ICC = 0.949; 95% CI: 0.870–0.981).

### 3.2. Participant Characteristics and Study Flow

Participant characteristics for all enrolled participants (*n* = 15) are presented in Table 1. All participants completed each of the four experimental conditions and were included in the final analysis.

**Figure 1:**
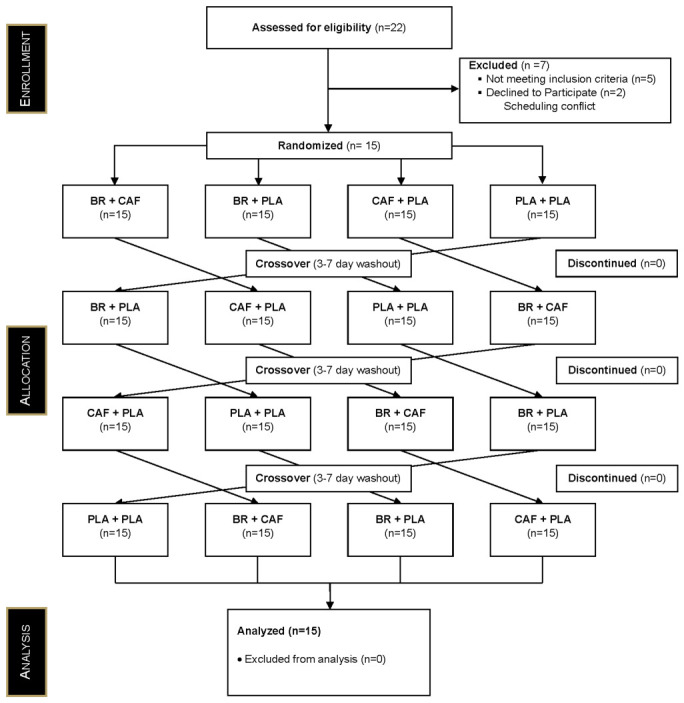
CONSORT diagram demonstrating participant flow.

A total of 22 individuals were screened for eligibility. Seven participants were excluded prior to enrollment, with five of these individuals not meeting the inclusion criteria and two who declined participation due to scheduling conflicts. Fifteen participants were randomized and completed all study visits. The Consolidated Standards of Reporting Trials (CONSORT) flow diagram is presented in Figure 1. No adverse events were reported throughout the study.

### 3.3. Dietary Intake

Self-reported relative energy and relative macronutrient intake preceding each trial were comparable across conditions (Table 2).

### 3.4. Exercise Performance

Performance outcomes are presented in Table 3. There was no significant effect of condition on EP (*p* = 0.401, ηp^2^ = 0.056) or WEP (*p* = 0.580, ηp^2^ = 0.048). Mean EP values were 282.2 W (95% CI: 257.1–307.9) for PLA, 272.0 W (227.2–316.8) for BR, 280.1 W (254.5–305.6) for CAF, and 289.9 W (265.1–314.7) for BR + CAF. Corresponding WEP values were 15.17 kJ (95% CI: 11.74–18.60) for PLA, 13.85 kJ (10.14–17.56) for BR, 14.77 kJ (9.78–19.75) for CAF, and 14.02 kJ (10.25–17.78) for BR + CAF. There was no significant main effect of period for EP (*p* = 0.578) or WEP (*p* = 0.549), suggesting that trial order did not meaningfully influence performance outcomes.

**Figure 2:**
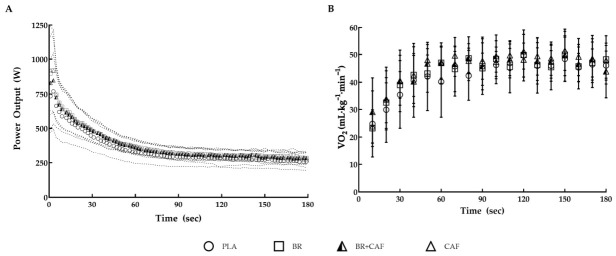
Effects of placebo, beetroot juice, caffeine, and beetroot juice plus caffeine on mean power output (**A**) and oxygen uptake (VO_2_) (**B**) during a 3 min all-out cycling test. In panel (**A**), dashed lines represent the standard deviation. In panel (**B**), the VO_2_ plateau was reached at approximately 60 s.

Similarly, peak power (*p* = 0.642, ηp^2^ = 0.046), mean power (*p* = 0.212, ηp^2^ = 0.108), and total work (*p* = 0.217, ηp^2^ = 0.107) obtained during the 3MT were not significantly different between conditions. Average power output across time intervals demonstrated a similar pattern, with values remaining closely aligned between conditions. At 60 s, the condition effect was not statistically significant (*p* = 0.069, ηp^2^ = 0.164). At all other timepoints, average power output was comparable between conditions, including 30 s (*p* = 0.432, ηp^2^ = 0.067), 90 s (*p =* 0.770, ηp^2^ = 0.028), 120 s (*p* = 0.350, ηp^2^ = 0.080), and 150 s (*p* = 0.148, ηp^2^ = 0.127) (Figure 2A). Oxygen uptake increased rapidly following the onset of the test and reached a plateau in all conditions. No significant main effect of condition was detected for VO_2_peak (*p* = 0.246, ηp^2^ = 0.112) (Figure 2B).

## 4. Discussion

The purpose of the present study was to examine the effects of acute dietary nitrate and caffeine supplementation on performance outcomes derived from a 3 min all-out (3MT) cycling test in trained male cyclists. The principal finding was that no significant differences were observed between conditions (PLA, BR, CAF, and BR + CAF) for EP or WEP, which were used as surrogate measures of critical power and *W*′, respectively. To our knowledge, this is the first study to investigate these supplementation conditions using the 3MT protocol in a trained population. These findings suggest that, under the present dosing conditions and within the 3MT model, acute supplementation did not produce detectable differences in performance outcomes in trained male cyclists.

Several studies have previously examined the combined effects of dietary nitrate and caffeine on endurance performance using a range of exercise models, with mixed findings. Handzlik et al. [23] reported nonsignificant but potentially meaningful improvements in time to exhaustion following combined supplementation during prolonged cycling, which the authors attributed primarily to reductions in perceived exertion rather than clear physiological enhancements. In contrast, Lane et al. [24] observed improved performance following caffeine ingestion alone during a 20 km cycling time trial in trained female cyclists, with no additional benefit when caffeine was combined with nitrate. Similarly, Oskarsson et al. [25] showed no significant effects of combined nitrate and caffeine supplementation on either submaximal running performance or a maximal 1 km time trial. Collectively, these findings suggest that performance outcomes are not consistently improved across different supplementation conditions, although results remain dependent on the exercise model and population studied.

In contrast to the limited evidence for additive effects when combined, both dietary nitrate and caffeine have substantial support for their ergogenic potential across a range of exercise models. Dietary nitrate supplementation has been shown to enhance exercise efficiency during submaximal exercise [26], increase tolerance to severe-intensity work [6], and, in some cases, improve performance in time-to-exhaustion [3] and time-trial protocols [4]. However, effects on critical power-related outcomes appear to be small and inconsistent, particularly in trained populations. For example, Kelly et al. [18] reported improvements in exercise tolerance across several severe-intensity constant load trials following nitrate supplementation, yet did not observe statistically significant changes in estimated critical power or *W*′. This pattern is consistent with the broader literature, as highlighted in a recent meta-analysis by Senefeld et al. [27], which reported that the overall effect size of nitrate supplementation is small, with a substantial proportion of studies failing to detect statistically significant performance benefits. Small changes in performance have nevertheless been reported in some investigations despite the absence of statistical significance. Whether such differences translate to meaningful advantages depends on the competitive context, measurement precision, and the reliability of the assessment. Importantly, even when statistical significance is not achieved, modest performance improvements have been reported and may still be meaningful for competitive athletes. For instance, Lansley et al. [6] observed no differences in finishing time during a 16.1 km cycling time-trial despite a reduction in completion time relative to placebo. Even from this perspective, the magnitude of difference between conditions failed to suggest that consistent but small changes were occurring in one treatment versus the other.

Caffeine supplementation is also well established as an ergogenic aid and remains one of the most widely consumed supplements among competitive athletes [2]. Although most caffeine research in endurance performance has focused on time-trial and time-to-exhaustion protocols, Cheng et al. [28] employed a 3 min all-out cycling test and reported that ingestion of 6∙mg∙kg^−1^ caffeine increased WEP in well-trained cyclists, while EP itself was unaffected. The authors suggested that the high-intensity nature of the test may have amplified caffeine’s central nervous system effects, potentially reducing perceived exertion during the latter portion of the effort, thereby enabling participants to sustain higher power output for a longer duration prior to reaching end power. These findings are consistent with earlier work by Simmonds et al. [29], who demonstrated that caffeine increased maximal accumulated oxygen deficit, a variable associated with anaerobic work capacity. From a physiological perspective, the combined administration of nitrate and caffeine may not influence the primary determinants of performance within the same task demands and time window. During a 3MT, performance is thought to progress from an initial phase characterized by very high neuromuscular demands, toward a period dominated by severe-intensity metabolic disturbance, before ultimately stabilizing near an end-test power plateau, as described in the 3MT model [16,21]. If nitrate supplementation primarily influences oxygen delivery, mitochondrial efficiency, or the oxygen cost of exercise during longer or steadier efforts, its capacity to modify outcomes during a brief maximal protocol such as the 3MT may be limited, particularly compared to exercise models involving longer-duration, steady-state, or time-trial efforts where nitrate supplementation has more consistently demonstrated ergogenic effects [30]. Accordingly, the absence of an observed effect in the present study should be interpreted within the context of this potential mismatch between the physiological targets of nitrate supplementation and the characteristics of the exercise model.

In addition, physiological responses to the BR + CAF condition may be complex and not necessarily complementary. Dietary nitrate supplementation increases nitric oxide bioavailability, promoting vasodilation and potentially enhancing muscle blood flow and oxygen delivery. In contrast, caffeine acts as an adenosine receptor antagonist and may increase sympathetic activity, which can result in vasoconstrictive effects in certain vascular beds. As such, these opposing peripheral influences, combined with caffeine’s centrally mediated effects on arousal and perceived exertion, may not align within the same physiological pathways or time course to produce additive effects during a short-duration, high-intensity task. Nevertheless, in the present cohort, these influences were insufficient to produce detectable changes in EP or WEP. Some time-specific responses were associated with moderate to large effect sizes despite the absence of statistical significance. In the context of the modest sample size, these findings may reflect potentially meaningful effects that the present study was underpowered to detect and should therefore be interpreted with appropriate caution.

Possible explanations for the absence of an ergogenic effect in the present study are likely multifactorial. Previous work has suggested that the performance benefits of dietary nitrate supplementation may be attenuated as aerobic fitness increases, potentially due to adaptations such as greater endogenous nitric oxide availability, enhanced muscle capillarization, and improved regulation of oxygen delivery and utilization [8,27]. The participants in the present investigation were classified as trained rather than well-trained or elite [20], and therefore, training status may have contributed, but does not fully explain the absence of effect. Instead, the results may reflect the generally small magnitude of effect associated with nitrate supplementation, combined with normal biological variability in responsiveness among individuals.

Training status alone does not fully account for the absence of a caffeine effect. Caffeine has previously been shown to enhance WEP in well-trained cyclists, and some evidence suggests that trained individuals may experience equal or even greater benefits compared with untrained populations. It should also be noted that participants in the present study were classified as trained rather than highly trained or elite, which limits the extent to which these findings can be generalized to higher-performance populations. Taken together, these observations suggest that inter-individual variability, rather than a single characteristic such as training status, may have influenced the response to supplementation in the present study. Both caffeine and dietary nitrate are known to exhibit substantial inter-individual variability in ergogenic response. Variability in caffeine responsiveness has been linked to factors such as genetic polymorphisms (e.g., CYP1A2) [31], while the influence of habitual caffeine intake remains equivocal, with current evidence suggesting inconsistent effects on acute performance responses [9,32]. Similarly, variability in nitrate responsiveness may be influenced by differences in nitrate–nitrite–nitric oxide metabolism and oral microbiome activity [30]. In the absence of control for these factors, heterogeneous responses across participants may have attenuated group-level effects and contributed to the lack of statistically significant differences observed in the present study.

Key strengths of the present study included the randomized, double-blind, placebo-controlled, crossover design, which minimizes between-subject variability and enhances confidence in causal interpretation. The supplementation doses and timing were selected in accordance with the prior literature demonstrating ergogenic potential, and the exercise protocol employed has been previously validated for the estimation of performance outcomes related to critical power. Furthermore, participants completed two familiarization trials of the 3MT before experimental testing in an effort to increase the reliability of our findings while reducing the likelihood that learning effects influenced performance during the intervention visits. This approach appeared effective as test–retest reliability across familiarization trials was good to excellent for the primary performance outcomes (see Section 3.1).

Several limitations should be considered when interpreting the findings of this study. First, the trial was registered retrospectively at ClinicalTrials.gov following study completion, which should be considered a limitation related to transparency and study planning. Although the crossover design enhances statistical efficiency, the overall sample size was modest and may have limited the ability to detect moderate effects, increasing the risk of type II error. Accordingly, the absence of statistically significant differences should be interpreted with caution, as the study may have been underpowered to detect meaningful effects, and group mean responses may have masked potentially meaningful inter-individual variability. This is particularly relevant given the typically small effect sizes and substantial inter-individual variability associated with nitrate and caffeine supplementation. The study sample consisted exclusively of trained male cyclists, which may limit generalizability to female athletes, elite populations, or recreationally active individuals who may experience different physiological responses to supplementation. Future studies should include both sexes to improve external validity. In addition, nitrate was administered acutely, and these findings cannot be extrapolated to longer-term supplementation strategies. While the 3 min all-out test provides a practical and widely used estimate of CP and *W*′, it remains an indirect method and may be influenced by factors such as pacing strategy and individual variability. Dietary intake and adherence to pre-visit restrictions were based on self-report and could not be independently verified, which may have introduced variability despite efforts to standardize across trials. In addition, concentrations of plasma nitrate/nitrite or caffeine were not collected to confirm physiological responses to supplementation, limiting the ability to verify individual uptake and responsiveness. Finally, inter-individual variability in response to supplementation should be considered. Variability in caffeine responsiveness has been linked to habitual intake, genetic polymorphisms, and individual sensitivity, although evidence regarding the impact of habitual intake remains mixed [13]. Habitual caffeine intake was not controlled, and nitrate responsiveness was not assessed, which may have contributed to variability in performance responses and masked potential treatment effects. Future studies may benefit from accounting for these factors, as well as incorporating mixed-model approaches or individual response analyses, to better characterize variability in crossover designs.

## 5. Conclusions

No significant differences were observed between supplementation conditions for EP or WEP during a 3MT in trained male cyclists. However, given the modest sample size, these findings should be interpreted with caution, as the study may have been underpowered to detect meaningful effects. Accordingly, the results should be considered inconclusive rather than definitive evidence of no effect. Future research should continue to explore whether responses differ across populations, exercise models, or longer-term supplementation strategies.

## Figures and Tables

**Table 1:** Participant characteristics.

Variable	Mean ± SD
Age (years)	28 ± 5
Height (cm)	176.6 ± 7.7
Body Mass (kg)	79.2 ± 9.1
VO_2_peak (mL∙kg^−1^∙min^−1^)	55.2 ± 5.6
W_max_ (W)	380.7 ± 43.5
W_max_ (W∙kg^−1^)	4.8 ± 0.5
GET (% VO_2_peak)	68.6 ± 8.3
HR_peak_ (bpm)	188 ± 10

VO_2_peak = peak oxygen uptake; W_max_ = maximal power output; GET = gas exchange threshold; HR = heart rate; bpm = beats per minute; cm = centimeters; kg = kilograms; W = watts; W∙kg^−1^ = watts per kilogram; mL∙kg^−1^∙min^−1^ = milliliters per kilogram per minute; SD = standard deviation.

**Table 2:** Relative energy and macronutrient intake preceding each trial.

Variable	PLA	BR	CAF	BR + CAF	*p*
Energy (kcal·kg^−1^·day^−1^)	26.8 ± 13.3	31.7 ± 11.2	34.2 ± 11.6	32.8 ± 10.1	0.383
Carbohydrate (g·kg^−1^·day^−1^)	3.2 ± 1.7	3.6 ± 1.7	3.7 ± 1.5	3.6 ± 1.7	0.868
Protein (g·kg^−1^·day^−1^)	1.2 ± 0.6	1.4 ± 0.6	1.5 ± 0.7	1.4 ± 0.2	0.549
Fat (g·kg^−1^·day^−1^)	1.1 ± 0.7	1.4 ± 0.5	1.5 ± 0.4	1.5 ± 0.6	0.403

Data presented as mean ± SD; *p* = *p* value; PLA = placebo; BR = beetroot; CAF = caffeine; BR + CAF = beetroot plus caffeine; kcal·kg^−1^·day^−1^ = kilocalories per kilogram per day; g·kg^−1^·day^−1^ = grams per kilogram per day.

**Table 3:** Exercise performance variables.

Variable	PLA	BR	CAF	BR + CAF	*p*	ηp^2^
EP (W)	282 ± 46	272 ± 80	280 ± 46	290 ± 45	0.401	0.056
WEP (kJ)	15 ± 6	13 ± 6	14 ± 9	14 ± 7	0.580	0.048
Peak Power (W)	931 ± 322	920 ± 322	968 ± 316	909 ± 364	0.642	0.046
Mean Power (W)	374 ± 59	372 ± 58	375 ± 58	378 ± 57	0.212	0.108
Total Work (kJ)	67.4 ± 10.7	67.0 ± 10.5	67.6 ± 10.5	68.0 ± 10.4	0.217	0.107
PO_30_ (W)	610 ± 136	597 ± 134	618 ± 125	606 ± 150	0.432	0.067
PO_60_ (W)	408 ± 60	411 ± 63	424 ± 64	418 ± 63	0.069	0.164
PO_90_ (W)	334 ± 52	334 ± 51	337 ± 47	337 ± 46	0.770	0.028
PO_120_ (W)	311 ± 44	307 ± 50	303 ± 51	311 ± 39	0.350	0.080
PO_150_ (W)	299 ± 46	296 ± 44	290 ± 56	304 ± 41	0.148	0.127

Data presented as mean ± SD; *p* = *p*-value; ηp^2^ = partial eta squared; PLA = placebo; BR = beetroot; CAF = caffeine; BR + CAF = beetroot plus caffeine; EP = end-test power output; WEP = work done above end-test power output; PO_30_ = averaged power output from 0 to 30 s, PO_60_ = averaged power output from 0 to 60 s; PO_90_ = averaged power output from 0 to 90 s; PO_120_ = averaged power output from 0 to 120 s; PO_150_ = averaged power output from 0 to 150 s; W = watts; kJ = kilojoules.

## Data Availability

The datasets used and/or analyzed during the current study are available from the corresponding author upon reasonable request.

## Abbreviations

The following abbreviations are used in this manuscript:

ANOVA	Analysis of variance
BR	Beetroot juice
BR + CAF	Beetroot juice plus caffeine
CAF	Caffeine
CP	Critical power
EP	End power
GET	Gas exchange threshold
HR	Heart rate
HR_peak_	Peak heart rate
ICC	Intraclass correlation coefficient
PLA	Placebo
*V_E_*/VO_2_	Ventilatory equivalent for oxygen
*V_E_*/VCO_2_	Ventilatory equivalent for carbon dioxide
VO_2_peak	Peak oxygen uptake
WEP	Work above end power
*W*′	Curvature constant of the power-duration relationship
3MT	3 min all-out test
ηp^2^	Partial eta squared
